# Infratemporal Space Infection Following Maxillary Third Molar Extraction in an Uncontrolled Diabetic Patient

**DOI:** 10.5681/joddd.2012.024

**Published:** 2012-09-01

**Authors:** Ali Hossein Mesgarzadeh, Mohammad Ali Ghavimi, Gulşen Gok, Afsaneh Zarghami

**Affiliations:** ^1^Associate Professor, Department of Oral and Maxillofacial Surgery, Faculty of Dentistry, Tabriz University of Medical Sciences, Tabriz, Iran; ^2^Assistant Professor, Department of Oral and Maxillofacial Surgery, Faculty of Dentistry, Tabriz University of Medical Sciences, Tabriz, Iran; ^3^Post-graduate Student, Department of Oral and Maxillofacial Surgery, Eastbourne District General Hospital, East Sussex, England; ^4^Undergraduate Student, Faculty of Dentistry, Tabriz University of Medical Sciences, Tabriz, Iran

**Keywords:** Diabetes mellitus, infratemporal space, odontogenic infection

## Abstract

Infratemporal space infection is a rare but serious sequel of odontogenic infection. The diagnosis is difficult due to non spe-cific signs and symptoms. Diabetes mellitus as a definitive risk factor for odontogenic infections needs more consideration during clinical procedures. We report a case of an undiagnosed diabetic patient with isolated infratemporal space infection after tooth extraction with presentation of similar signs and symptoms of temporomandibular joint and muscle problem.

## Introduction

The infratemporal fossa is an anatomic space of great importance in the head.^1 - 3^ Abscesses of this space are rare but potentially life threatening. With regard to the proximity to some important anatomical areas of the head, dealing with infratemporal space infection needs great consideration both in examination and surgical practice.^1 , 4^The infection might spread through the pterygoid plexus to the cavernous sinus or through the valveless ophthalmic veins into the orbit.^5 - 7^Isolated infection of the infratemporal space is rare and difficult to diagnose.,^4^ Clinical symptoms of pain, trismus, and fever are more likely to be diagnosed as a joint or muscle disorder.^1 , 5^

To the best of our knowledge, a few cases of the diagnostic dilemma have been reported in the literature. Therefore, we aimed to report a case of isolated infratemporal space infection after extraction of a maxillary third molar in an uncontrolled diabetic patient that had been misdiagnosed as a temporomandibular joint (TMJ) disorder.

## Case report

A 40-year-old male with the chief complaint of pain on the right side of the face and slight swelling in condylar area following a complicated maxillary right third molar extraction 24 days before, was referred to the Department of Oral and Maxillofacial Surgery, Tabriz University of Medical Sciences. Trismus and tenderness was observed in the right condylar area. Symptoms of pain and trismus had started five days after extraction. Other presentations were dysphagia, odynophagia, chills, and fever (39ºC).

The condition had been diagnosed and treated as a dry socket initially. However, prescription of amoxicillin 500 mg three times a day for a week had not improved the symptoms. Therefore, treatment of TMJ dysfunction syndrome by moist heat pack, muscle relaxant and NSAIDS was started. Pain, click and trismus had gradually risen.

Past medical history showed no systemic diseases; however, the patient complained of polyuria and sudden weight-gaining since six months ago. The history of chronic myofacial pain and TMJ dysfunction syndrome and long-term appliance therapy was remarkable with the complaint of recurrent periods of trismus and tenderness on the right condylar area and facial muscles since two years ago. The patient was using a night guard appliance in periods of recurrent and uncontrollable pain due to bruxism and clenching.

Extraoral examination indicated slight swelling and tenderness on the right side of the face with non-pitting appearance on palpation of the jaw. Right parotid gland was non-tender. No cervical lymphadenopathy was noted. Otologic and rhinoscopic examinations were unremarkable.

Slight tenderness without purulent discharge was observed around the extraction socket of right third molar. Posterior open bite was remarkable on the right side of centric occlusion. Trismus with maximum interincisal distance less than 15 mm was observed. Needle aspiration of the area was non-productive.

An axial and coronal spiral computed tomography (CT) scan with 5 mm sections on the condyle and infratemporal fossa revealed right infratemporal space cellulitis with accumulation of the fluid in right TMJ (Figure 1).


Figure 1. Coronal CT scan shows right infratemporal space celullitis (A); axial view (B).

**A:**
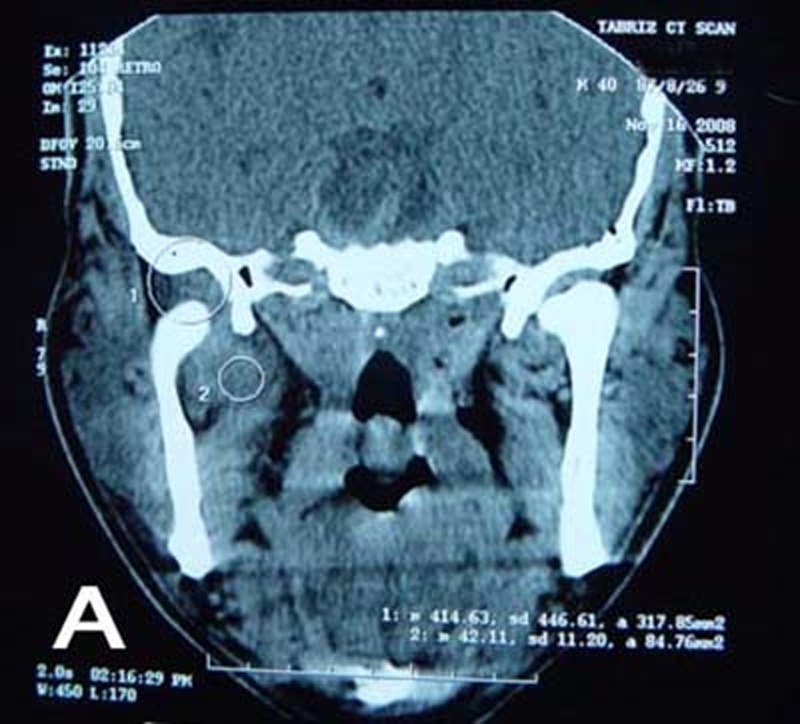

**B:**
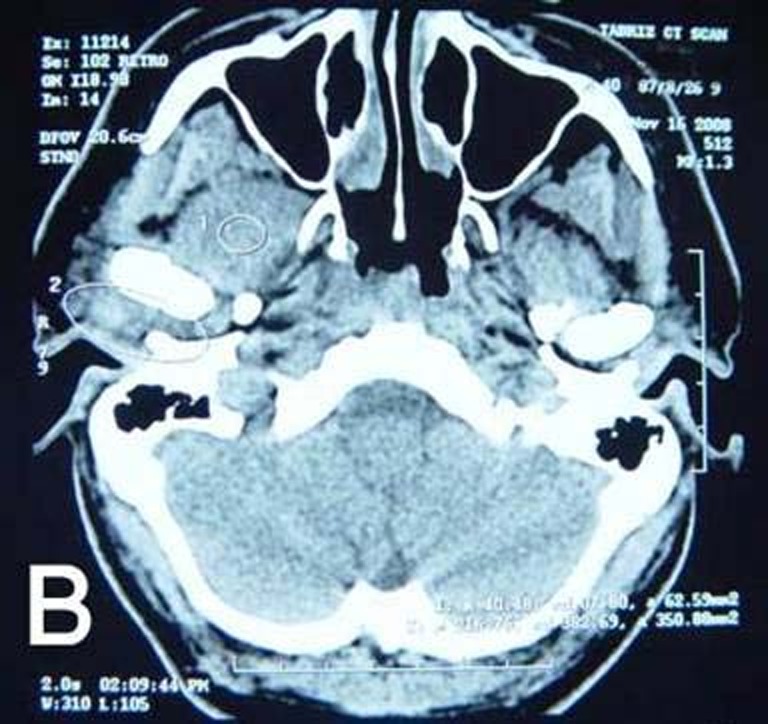

The patient was admitted to the hospital immediately and empirical intravenous antibiotic therapy with penicillin and metronidazole was started. Serum fasting blood sugar (FBS) was checked twice, which the results were more than 196 mg/dl and glucose tolerance test (GTT) was 250 mg/dl. Consulting with the internal medicine service, insulin therapy was started to control blood glucose level. Empirical antibiotic therapy improved trismus, and maximum interincisal distance reached to 25 mm.

The patient was taken to the operating room for intraoral incision and drainage. Under endoscopic nasal intubation and general anesthesia, intra-oral incision over the anterior aspect of the ascending ramus was carried out and dissection was continued to the condylar and infratemporal area. As soon as the hemostat reached to the infratemporal space pus began to flow. Opening the hemostat along with moving mandible caused further drainage. Drain was placed in the site. The result of microbiological culture was unremarkable.

**Figure 2:**
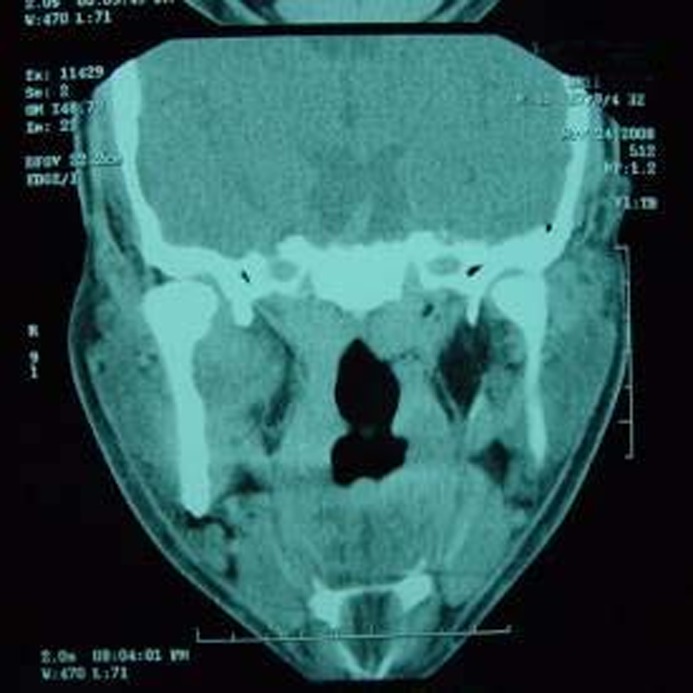
In postoperative CT, edema has disappeared.

The patient complained of severe headache and vomiting after surgery, however, previous signs and symptoms were improved. Consulting with neurosurgery service, CT scan of brain ruled out brain abscess formation. Drain removed three days after surgery. Post operative CT scan showed normal tissue structure (Figure 2). The patient was discharged from hospital with oral antibiotics for further 7 days.

## Discussion

Infratemporal fossa infection occurs secondary to odontogenic infections with the common origin of mandibular molars.^6 , 7^ Abscesses are rare and potentially dangerous complications. Besides, clinical diagnosis tends to be challenging as a result of non-specific signs and symptoms.,^4^ Different manifestations might be observed depending on the specific anatomical feature involved in the infection including pain, fever, trismus in acute infection and trismus with swelling in chronic infection and even neurosensory deficit.^8 - 10^Trismus can be the diagnostic hallmark to distinguish infratemporal space infection from other conditions with facial swelling.

The differential diagnosis may include parotitis and temporomandibular joint disorder.^1^ Considering past medical and dental history, clinicians should perform an accurate intra- and extra-oral examination in order to get to a proper diagnosis. Furthermore, the precise information of imaging modalities especially CT scan will allow for timelier and targeted diagnosis and treatment.^11^ CT scan is known as the only way to detect characteristic signs such as lucency and gas bubbles definitively.^12^

In agreement with most reported cases, microbial culturing showed no particular organism isolation. In addition to the polymicrobial nature of odontogenic infections, the reason might be several antibiotic prescriptions before final diagnosis.^1^

Clinical management of the patient with serious infection needs complete information about the systemic condition. In immunocompromised patients, such as uncontrolled diabetes mellitus (DM), proper diagnosis as well as prompt surgical intervention is critical in emergency situations. Before the surgical procedure, standard antibiotic prophylaxis and medical supervision are essential for these patients. It is necessary to consider that empirical antibiotic therapy should cover Klebsiella pneumonia in diabetic patients. ^10^ The treatment should focus on prevention of spreading infection with possible life-threatening complications.
